# Influences of Different Large Mammalian Fauna on Dung Beetle Diversity in Beech Forests

**DOI:** 10.1673/031.013.5401

**Published:** 2013-06-14

**Authors:** Hiroto Enari, Shinsuke Koike, Haruka Sakamaki

**Affiliations:** 1 Satoyama Science Research Center, Faculty of Agriculture, Utsunomiya University, Minemachi 350, Utsunomiya, Tochigi 321-8505, Japan; 2 Institute of Agriculture, Tokyo University of Agriculture and Technology, 3-5-8 Saiwaicho, Fuchu, Tokyo 183-8509, Japan; 3 The United Graduate School of Agricultural Sciences, Iwate University, 3-18-8 Ueda, Morioka, Iwate 020-8550, Japan; 4 Present address: Faculty of Agriculture, Yamagata University, 1-23 Wakabamachi, Yamagata 997-8555, Japan

**Keywords:** biological interaction, cool-temperate forest, species diversity

## Abstract

This paper focuses on biological relationships between mammalian species richness and the community structure of dung beetles in cool-temperate forests in the northernmost part of mainland Japan. The composition of beetle assemblages was evaluated at 3 sites in undisturbed beech forests with different mammalian fauna. In spring and summer 2009, beetles were collected at each site using pitfall traps baited with feces from Japanese macaques, *Macaca fuscata* Blyth (Primates: Cercopithecidae); Asiatic black bears, *Ursus thibetanus* Cuvier (Carnivora: Ursidae); Japanese serows, *Capricornis crispus* Temminck (Artiodactyla: Bovidae); and cattle. In the present study, 1,862 dung beetles representing 14 species were collected, and most dung beetles possessed the ecological characteristic of selecting specific mammalian feces. The present findings indicated that although species diversity in dung beetle assemblages was not necessarily positively correlated with mammalian species richness in cool-temperate forests, the absence of the macaque population directly resulted in the marked reduction of the beetle abundance, with the loss of the most frequent species, *Aphodius eccoptus* Bates (Coleoptera: Scarabaeidae) during spring.

## Introduction

In most terrestrial ecosystems, dung beetles perform key ecological functions, such as nutrient recycling, parasite suppression, and secondary seed dispersal (Nichols et al. 2008). Dung beetle diversity and composition are highly sensitive to the availability of essential resources (i.e., feces), and the abundance and distribution of resource providers (i.e., mammals) are considered a key determinant of the community structure of dung beetles (Hanski and Cambefort 1991a; Nichols et al. 2008, 2009). The relationship between mammalian species richness and the community structure of dung beetles has been addressed in several empirical studies (Estrada et al. 1998, 1999; Feer and Hingrat 2005; Harvey et al. 2006; Andresen and Laurance 2007); however, these studies have been conducted only in tropical landscapes. Other landscape types, such as high-latitude forests, where species richness of the beetle is naturally low (Hanski and Cambefort 1991a; Davis et al. 2002) have not been investigated. Moreover, only limited studies have documented the potential effects of changes in mammal communities on dung beetle assemblages distinct from other environmental changes such as habitat modification (Andresen and Laurance 2007; see also review paper by Nichols et al. 2009).

**Figure 1.**
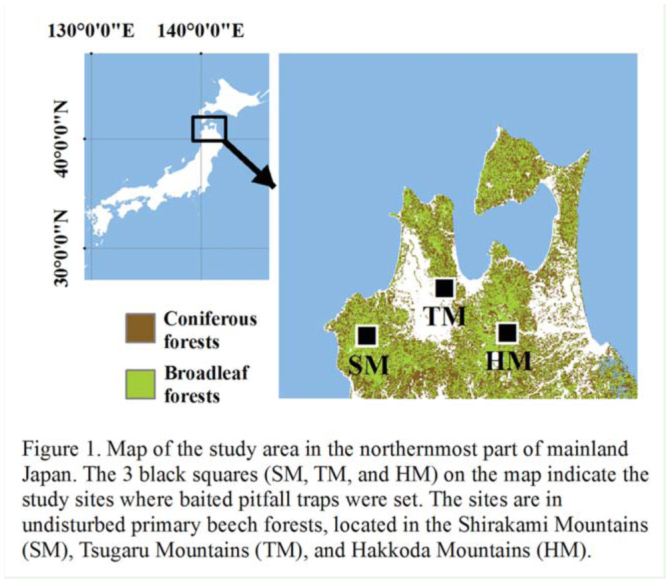
Map of the study area in the northernmost part of mainland Japan. The 3 black squares (SM, TM, and HM) on the map indicate the study sites where baited pitfall traps were set. The sites were in undisturbed beech forests, located in the Shirakami Mountains (SM), Tsugaru Mountains (TM), and Hakkoda Mountains (HM). High quality figures are available online.

The purpose of this study was to identify the biological influences of different mammal faunas on the diversity and composition of dung beetle assemblages in the cool-temperate forests of Aomori Prefecture, located in the northernmost part of mainland Japan (Figure 1). Large mammalian fauna vary widely within this forested region because of past local extinctions (Biodiversity Center of Japan 2010); most large mammals were locally overhunted for food, medicines, and mammalian pest control before the Second World War (Mito 1997; Tsujino et al. 2010). Apart from the availability of feces, most dung beetle species are highly vulnerable to human-induced habitat disturbance, such as deforestation, which leads to changes in local microclimates and microhabitats (Davis et al. 2001; Estrada and Coates-Estrada 2002; Halffter and Arellano 2002; Nichols et al. 2007; Navarrete and Halffter 2008). Therefore, to eliminate anthropogenic influences on the local natural vegetation and landscape structure except existing local mammalian fauna, 3 study sites were selected within the same forest-landscape of undisturbed beech, *Fagus crenata* Blume (Fagales: Fagaceae), forest, which is one of several representative forest types of the Palearctic region. In 2009, the diversity and composition of dung beetles at each site were evaluated using pitfall traps baited with mammalian feces. Some dung beetle species show clear selectivity for the feces of different animals according to size and contents (Fincher et al. 1970; Hanski and Cambefort 1991a; Martin-Piera and Lobo 1996), but descriptive information about the feces preference of dung beetles inhabiting cool-temperate forests is fragmented (Kawai et al. 2005). Therefore, the feces of Japanese macaques, *Macaca fuscata* Blyth (Primates: Cercopithecidae), Asiatic black bears, *Ursus thibetanus* Cuvier (Carnivora: Ursidae), Japanese serows, *Capricornis crispus* Temminck (Artiodactyla: Bovidae), cattle (typified by grassland mammals) were used as bait in the pitfall traps. This sampling regime was used to address 2 questions: (1) Is each dung beetle species dependent on a specific type of mammalian feces? (2) Is species diversity in dung beetle assemblages positively correlated with mammalian species richness in cool-temperate forests?

## Materials and Methods

### Study area

Nearly 70% of Aomori Prefecture is covered by cool-temperate forests (Figure 1) composed of mainly beech and oak (*Quercus crispula* Blume) trees as well as coniferous plantations of Japanese cedar (*Cryptomeria japonica* D.Don) and Japanese larch (*Larix kaempferi* Carr.). Since most of these forested areas are mountainous, human settlements are concentrated in the narrow flatlands along rivers. The area is in the cool-temperate climatic zone. Mean air temperature and annual precipitation in 2009 were 10.6° C and 1,177 mm, respectively (Japan Meteorological Agency 2010). The period of snowfall was from early December to late March, with a maximum snow depth of around 2 m in lowland forested areas and 3–5 m in mountainous forested areas. Lingering snow was observed until late May.

Seven species of medium- and large-sized mammals—Japanese serows, Japanese martens (*Martes melampus* Wagner), raccoon dogs (*Nyctereutes procyonoides* Gray), Japanese badgers (*Meles anakuma* Temminck), Japanese weasels *(Mustela itatsi* Temminck), red foxes (*Vulpes vulpes* Linnaeus), and Japanese hares (*Lepus brachyurus* Temminck)— have ranged throughout the prefecture (Biodiversity Center of Japan 2010). However, past overhunting and human destruction of native forests caused the extinction of populations of gray wolves (*Canis lupus* Linnaeus), sika deers (*Cervus nippon* Temminck), and wild boars (*Sus scrofa* Linnaeus) in this prefecture, and the distributions of Japanese macaques and Asiatic black bears are still fragmented and isolated (Mito 1992, 1997; Tsujino et al. 2010).

### Study animals

Dung beetles are classified as part of the superfamily Scarabaeoidea and use animal excrement and carcasses as food or oviposition resources. Thus far, 152 species and 9 subspecies of indigenous dung beetles have been confirmed in the Japanese archipelago (Kawai et al. 2005). Dung beetles are generally divided into 3 groups according to nesting strategy: tunnellers, dwellers, and rollers (Cambefort and Hanski 1991). Most dung beetle species inhabiting Japan are classified as tunnellers or dwellers (Kawai et al. 2005).

In the past, scientific literature regarding the ecology and society of forest dung beetles in the cool-temperate zones of northern Japan has been limited. Recently, however, Enari et al. (2011) evaluated the community structure of dung beetle species using the feces of Japanese macaques in similar study areas. They confirmed (1) the presence of 14 dung beetle species comprising 8 dwellers and 6 tunnellers, and (2) that the frequency of occurrence of every beetle species was high in spring and low in autumn.

### Dung beetle sampling

Dung beetles were sampled using baited pitfall traps in spring (mid-June; i.e., soon after the snow melted) and summer (mid-August; i.e., the warmest period) 2009, but not in autumn and winter, which are the inactive seasons for dung beetles in cool-temperate forests (Enari et al. 2011). The traps were set in the following 3 sites within the study area: the southern Tsugaru Mountains (TM), which are classified as a Special Protected Zone of a Wildlife Protection Area; the southeastern Hakkoda Mountains (HM), which contain national parks with Special Zone Protection status; and the northeastern Shirakami Mountains (SM), where primary forests are listed as a World Nature Heritage site by UNESCO (Figure 1). These 3 sites are commonly covered with undisturbed beech forests, but compositions of large mammalian species are different today than they were historically (Table 1). Mean elevation and mean canopy openness, calculated using CanopOn 2.03 (Takenaka 2009) with hemispherical photographs, are about the same among the 3 sites (Table 1). Mean air temperature during the study period ranged from 14.5 to 17.2° C in spring and from 20.2 to 23.0° C in summer (Table 1).

Four parallel 40-m line transects at 20-m intervals were established at each site, and 5 sampling points at 10-m intervals along each transect were set. Then, pitfall traps, made from plastic containers (14 cm in diameter and 10 cm deep) and plastic cups (8 cm in diameter and 5 cm deep), were set at each sampling point. Trap design was based on that of Enari et al. (2011). Each container holding 50% ethylene glycol (used as a preserving fluid) was buried to its rim in the ground, and the cup was hung by a wire in each container. A sample of fresh feces (15 g) from each one of 4 mammals—Japanese macaques, Asiatic black bears, Japanese serows, and cattle—was placed into the cups equipped in the respective trap sets along the transect (i.e., 5 traps per each mammal feces at every site). To acquire fresh feces from wild macaques, macaque troops in the area around the SM study sites were followed. Fresh feces from wild bears were acquired from bears that were captured in box traps in the same area. Serow feces were collected from a neighboring zoo because of the difficulty in accessing fresh serow feces. Fresh cattle feces were obtained from a nearby cowshed. The feces samples were homogeneously mixed for use in the pitfall traps. A preliminary examination of the contents of each mammalian feces using the point quadrat method (Stewart 1967) showed variations among different mammal feces and seasonal changes, except in cattle feces (Table 2).

Traps were checked 24 and 48 hours after deployment each season, and all trapped dung beetles were collected. Voucher specimens were deposited at the Institute of Symbiotic Science and Technology, Tokyo University of Agriculture and Technology, Tokyo, Japan.

### Data analysis

First, to evaluate inter-sample variation in the composition of dung beetle assemblage within each trap set baited with mammalian feces at the 3 sites, the Friedman test was conducted for 10 samples, i.e., 5 sampling points × 2 terms (24 and 48 hours after deployment). Second, to evaluate the completeness of the dung beetle samples, the true species richness of each sample was estimated using the abundance-based coverage estimator (Chao and Lee 1992). This estimator uses species-bysample data and was only calculated when the total abundance captured was > 1. Furthermore, the estimator, which was a nonparametric richness estimator, assumed that the observed number of species in a sample comprised a number of abundant (common) and rare (infrequent) species. A cutoff value of 10 was used as the rare or infrequent boundary for the present study, as recommended by Chazdon et al. (1998). The software Estimates (Colwell 2006) was used to generate the abundance-based coverage estimator estimate with 100 randomizations without replacement.

**Figure 2.**
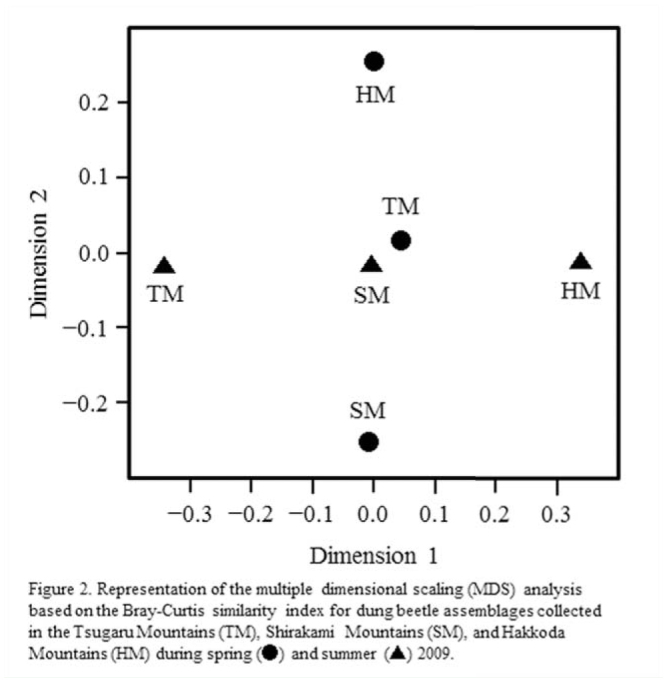
Representation of the multiple dimensional scaling analysis based on the Bray-Curtis similarity index for dung beetle assemblages collected in the Tsugaru Mountains (TM), Shirakami Mountains (SM), and Hakkoda Mountains (HM) during spring (circles) and summer (triangles) 2009. High quality figures are available online.

Since the sampling effort was the same at each sampling site, dung beetle species counts across sampling sites were compared using totals, percentages of occurrence, and inverse Simpson's diversity index (1/*D*) scores (Magurran 1988). Bonferroni *z*-statistics (Neu et al. 1974) was used to specify dominant species in the dung beetle assemblages collected at each location, assuming that all species appeared at the same occurrence rate. To evaluate the similarity in beetle assemblage compositions among sites, a multiple dimensional scaling analysis (Ludwig and Reynolds 1988) based on the Bray-Curtis similarity index (Bray and Curtis 1957) was used. In addition, the feces preference of each dung beetle species was evaluated using Bonferroni *z*-statistics. For each beetle species, it was assumed that the expected frequency of occurrence was the same for all types of mammalian feces.

## Results

During the spring and summer sampling at the 3 sites, 1,862 dung beetles were collected and 14 species composed of 7 tunnellers and 7 dwellers were confirmed. Compared with a unique inventory of dung beetle species in this region (Enari et al. 2011), 4 species were newly observed in the present study, while 5 species—all infrequent species with the exception of *Aphodius breviusculus* Motschulsky (Coleoptera: Scarabaeidae)— were not observed (Table 3). Both total abundance and total species richness of dung beetles observed in all trap sets were higher during spring (1,771 individuals representing 10 species) than summer (91 individuals representing 8 species). In addition, based on the values of inverse Simpson's diversity index, dung beetle assemblages collected in the TM showed the highest species diversity in both spring and summer. Results of the multiple dimensional scaling analysis for beetle assemblages trapped using the feces of the 4 mammals as bait clearly indicated that species compositions varied widely among sites in both spring and summer (Figure 2).

In spring, there was no significant intersample variation in the emerging pattern of dung beetles within each trap set, with the exception of traps that used bear feces and cattle feces in TM, and serow feces in HM (Friedman test: χ^2^ = 27.0, *p* = 0.001; χ^2^ = 18.9, *p* = 0.025; and χ^2^ = 25.2, *p* = 0.003, respectively), indicating that the sampling regime used was fairly robust. Mean completeness for the collections of beetles using pitfall traps in the TM, HM, and SM was 74.6%, 100.0%, and 71.7%, respectively; mean completeness was comparatively low for trap sets using bear and cattle feces in the TM because of the scarcity of the absolute abundance of beetles collected using those feces (Table 4). The abundance and species richness of dung beetles captured using each mammalian feces varied widely in spring. The abundance of beetles trapped using macaque feces was the highest at every site. However, the values of inverse Simpson's diversity index and the abundance of beetles captured did not necessarily show similar trends. Diversity was higher in the assemblages trapped using serow feces than in the assemblages using the other feces in both the HM and SM. The frequency of occurrence of individual species collected in spring showed a distinctive trend for *Aphodius eccoptus* Bates (Coleoptera: Scarabaeidae); abundance was notably high in the HM and SM, but was not observed in the TM.

**Figure 3.**
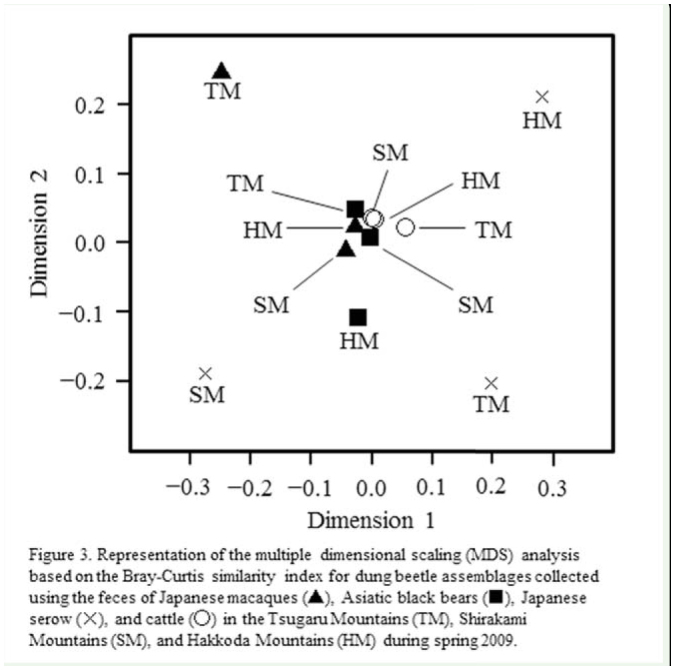
Representation of the multiple dimensional scaling analysis based on the Bray-Curtis similarity index for dung beetle assemblages collected using the feces of Japanese macaques (triangles), Asiatic black bears (squares), Japanese serow (exes), and cattle (circles) in the Tsugaru Mountains (TM), Shirakami Mountains (SM), and Hakkoda Mountains (HM) during spring 2009. High quality figures are available online.

When the similarity of dung beetle assemblages collected in spring was evaluated by multiple dimensional scaling analysis, the differences in species compositions among the 3 sites became more obvious (Figure 3). The analysis led to the following 3 findings: (1) species compositions of the assemblages using macaque feces were different in the TM than in the other 2 sites; (2) species compositions of the assemblages using bear and cattle feces showed no notable differences among the 3 sites; and (3) species compositions of the assemblages using serow feces showed large differences among the 3 sites.

In summer, there was no significant intersample variation in the emerging pattern of dung beetles within trap sets (Friedman test, *p* > 0.05 for all trap sets). Mean completeness for the collections of beetles in the TM, HM, and SM were 75.0%, 86.2%, and 79.2%, respectively (Table 5). Only the species *Phelotrupes laevistriatus* Motschulsky (Coleoptera: Geotrupidae) was commonly observed in every trap set at each site, except for the traps using cattle feces in the TM. The multiple dimensional scaling evaluation of the similarity of each assemblage collected in summer was omitted because of inadequate sample size.

Feces preference by dung beetle species during spring was evaluated. Summer data were not analyzed, due to insufficient sample size. Each dung beetle species clearly selected feces from a specific mammal (Table 6). In accordance with the Bonferroni *z*-statistics, 3 species, namely *Phelotrupes auratus* Motschulsky (Coleoptera: Geotrupidae), *P. laevistriatus*, and *A. eccoptus*, were highly attracted to macaque feces, and 3 species, namely *Onthophagus ater* Waterhouse (Coleoptera: Scarabaeidae), *Aphodius superatratus* Nomura, and *Aphodius unifasciatus* Nomura, intensively used serow feces. In particular, it was clear that *A. eccoptus* was heavily dependent on macaque feces. Neither bear nor cattle feces were attractive to any dung beetle species.

## Discussion

### Feces preference of dung beetle species

The results of our study clearly indicated that most dung beetles inhabiting undisturbed beech forests had the ecological characteristic of selecting a specific mammalian feces, as opposed to dung beetles inhabiting tropical landscapes; quite a few tropical dung beetles are considered generalists in their feeding preferences (Hanski and Cambefort 1991b). In particular, *A. eccoptus*, which was observed most frequently, is recognized as a specialist species that strongly prefers the feces of the Japanese macaque only. Since *A. eccoptus* was not observed in the TM, where the macaque population has been extinct for more than 100 years (Table 1), a reasonable conclusion is that the occurrence of *A. eccoptus* is highly dependent upon the availability of macaque feces.

However, during spring, the abundance of *A. eccoptus* was almost 3 times higher in the HM than in the SM, which is the only habitat where the macaque troops have continuously ranged (Table 4). This unexpected result might be explained as follows. First, macaque feces are available within the HM because non-troop solitary male macaques have frequented there (Oi et al. 1997; H. Enari, unpublished data; see Table 1), possibly allowing for the survival of the local population of *A. eccoptus* in the HM today. Second, the air temperature in the HM during spring was relatively low (because of its inland climate) compared with other 2 sites (Table 1) and could be more suitable for the outbreak of *A. eccoptus* in the forests. A massive outbreak of the beetle was observed in the SM at temperatures of around 10° C (Enari et al. 2011). To support these 2 explanations more empirically, further quantitative research on the minimum requirement of macaque feces for beetle reproduction would be required.

The results showing feces preference also demonstrated that few dung beetle species avoided using serow feces. This means that serow feces could be considered sharable resources for most dung beetle species, which resulted in the composition of dung beetle assemblages using serow feces differing between study sites (Figure 3). In contrast, bear and cattle feces were not attractive to most dung beetles (Table 6). In most study sites during both spring and summer, dung beetle species did not recognize cattle feces as a useable resource. This is possibly because cattle feces are not usually found in forest environments. The fundamental reasons for these fecal preferences are not fully understood from the present findings, but clearly cannot be explained by the contents of the respective mammalian feces alone. In fact, although there were no notable differences between the contents of bear and serow feces in spring or between serow and cattle feces in summer (Table 2), the beetle assemblages observed in each feces were not similar in composition. These facts most likely indicate that the digestive process of each mammal species, which potentially influences the fiber, nitrogenous, moisture, and volatile contents of feces, also affects the feces preference of dung beetles, as proposed by Davis (1989) and Davis and Scholtz (2001).

### Influences of mammalian species richness on dung beetle assemblages

In the collections captured using pitfall traps, 3 families representing 14 species were observed. All of the species except 2 are classified as forest species (Kawai et al. 2005); the remaining 2 species, *Aphodius uniformis* Waterhouse (Coleoptera: Scarabaeidae) and *Caccobius brevis* Waterhouse, which showed a low frequency of occurrence in the present study, range naturally in open-land habitats (Kawai et al. 2005). As with the study of cool-temperate dung beetles by Enari et al. (2011), the present data also demonstrated that most species showed obvious seasonal prevalence and emerged in abundance only during spring. One possible explanation for the massive appearance of dung beetles in spring might be the high availability of fresh feces. Feces were cryogenically preserved under the snow during winter and appeared on the ground as the snow melted in early spring. Further research on the resource value of such preserved feces for dung beetle species would be required to support this suggestion.

It is highly likely that the beetle community structure is sensitive to existing mammalian fauna, as evidenced by the fact that the species composition of assemblages varied widely with study site in both spring and summer (Figure 2). It should be noted that the species diversity of dung beetles was not positively correlated with the number of existing mammalian species; the beetle assemblages collected in the TM, which showed the most incomplete mammalian fauna among the study sites, demonstrated the highest species richness. This finding does not support previous studies (Estrada et al. 1999; Feer and Hingrat 2005; Andresen and Laurance 2007;Nichols et al. 2009), which commonly concluded that the population loss of mammalian species results in a decline in species richness of the beetles. The present result may be explained by the presence/absence of *A. eccoptus*, i.e., the most frequent species, which has also been shown to have high environmental tolerance (Enari et al. 2011). In sum, it is reasonable to consider that the massive occurrence of this species reduced the apparent values of inverse Simpson's diversity index in HM and SM during spring, owing to its statistical characteristics. When the index was recalculated for the spring dataset with *A. eccpotus* excluded, its value was 2.8 and 3.4 in HM and SM, respectively.

From the present findings, it may be concluded that although mammalian species richness is not necessarily positively correlated with species diversity in dung beetle assemblages in cool-temperate forests, the absence of the macaque population directly results in a marked reduction of the beetle abundance, as shown by the loss of *A. eccoptus*, which forms a commensal relationship with macaques during spring. To provide a better understanding of this exceptional conclusion in contrast to the previous studies (Nichols et al. 2009), further empirical research on the life-history traits and population dynamics of each dung beetle species would be required.

**Table 1.**
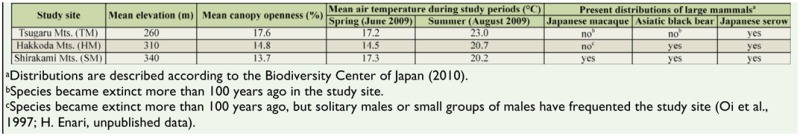
Environmental conditions (°C) and present distributions of large mammalian species in each study site located in the northernmost part of mainland Japan.

**Table 2.**
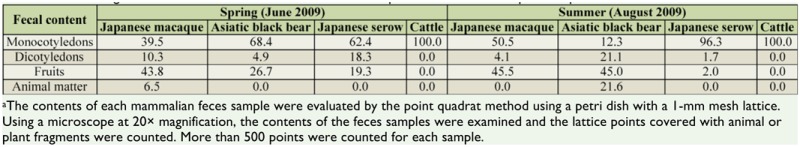
Percentage of the contents of each mammalian feces sample^a^ used in the baited pitfall traps.

**Table 3.**
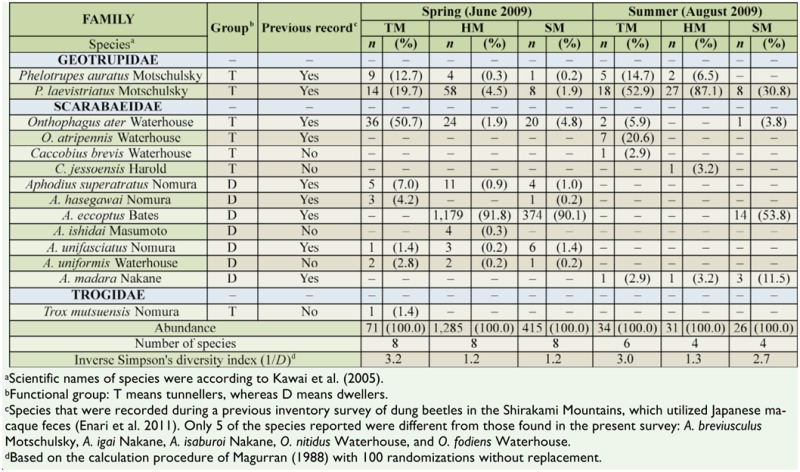
Regional and seasonal differences in the compositions of dung beetle species captured by pitfall traps baited with the feces of Japanese macaques, Asiatic black bears, Japanese serow, and cattle in primary beech forests, located in the Tsugaru Mountains (TM), Hakkoda Mountains (HM), and Shirakami Mountains (SM), northern Japan.

**Table 4.**
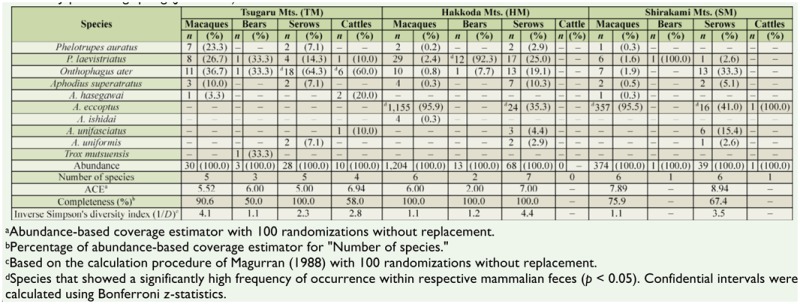
Diversity and composition of dung beetle species captured by pitfall traps baited with the feces of four large mammalian species—Japanese macaques, Asiatic black bears, Japanese serows, and cattles—in primary beech forests located in the northernmost part of mainland Japan during spring (June 2009).

**Table 5.**
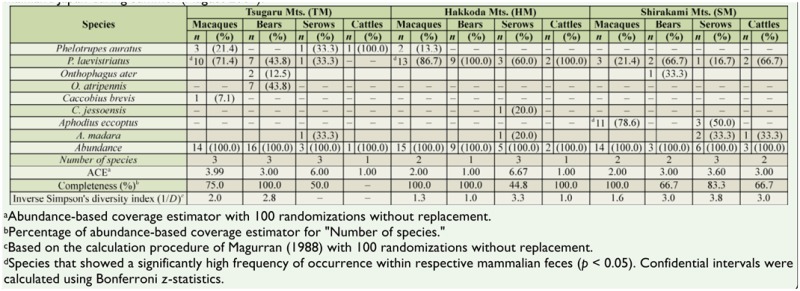
Diversity and composition of dung beetle species captured by pitfall traps baited with the feces of four large mammalian speciess—Japanese macaques, Asiatic black bears, Japanese serows, and catties—in primary beech forests located in the northernmost part of mainland Japan during summer (August 2009).

**Table 6.**
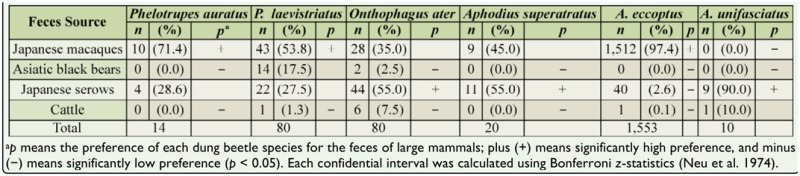
Preference of dung beetles (observed at > 10 individuals) for each mammalian feces, confirmed by baited pitfall traps in primary beech forests located in the northernmost part of mainland Japan during spring (June 2009). The data on occurrence frequency was pooled from the 3 study sites.

## Abbreviations

**HM**,: Hakkoda Mountains
**SM**,: Shirakami Mountains
**TM**,: Tsugaru Mountains
